# Supplementary material for: iRhom pseudoproteases regulate ER stress-induced cell death through IP3 receptors and BCL-2
Source: Nat Commun. 2022 Mar 10;13:1257. doi: 10.1038/s41467-022-28930-4 (PMC8913617; doi:10.1038/s41467-022-28930-4)
Supplement: Supplementary file 1 — Supplementary Information [file 41467_2022_28930_MOESM1_ESM.pdf]

iRhom pseudoproteases regulate ER stress-induced cell death through IP_3 receptors and BCL-2

Iqbal Dulloo[1*], Peace Atakpa-Adaji[2], Yi-Chun Yeh[3], Clémence Levet[1], Sonia Muliyil[1], Fangfang Lu[1], Colin W Taylor[2], and Matthew Freeman[1*]

[1]Dunn School of Pathology, University of Oxford, South Parks Road, Oxford OX1 3RE, UK

[2]Department of Pharmacology, University of Cambridge, Tennis Court Road, Cambridge, CB2 1PD, UK

[3]Department of Physiology, Anatomy and Genetics, University of Oxford, South Parks Road, Oxford, OX1 3PT, UK

[*] Corresponding authors: iqbal.dulloo@path.ox.ac.uk and matthew.freeman@path.ox.ac.uk

SUPPLEMENTARY FIGURES

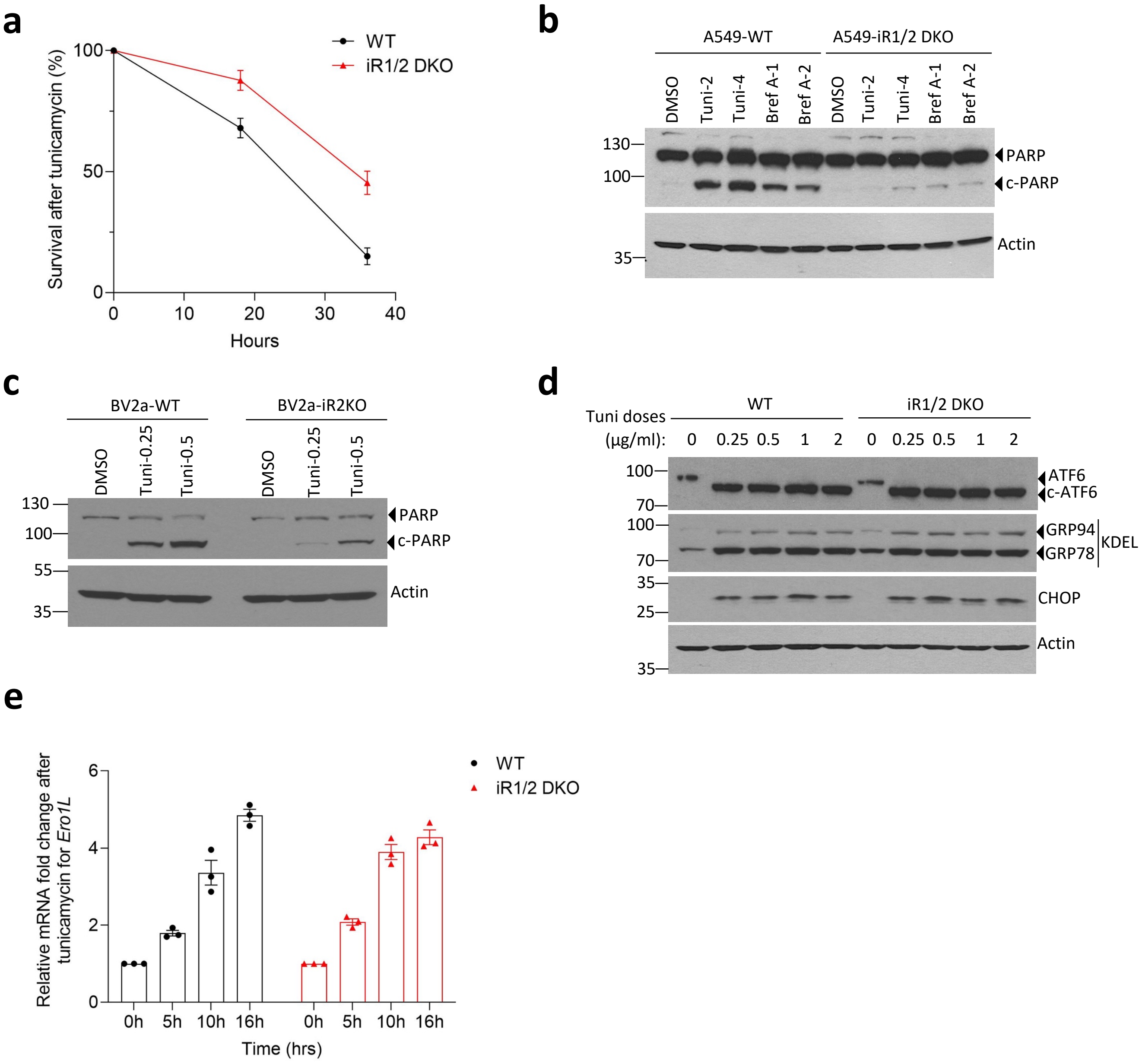

Supplementary Figure 1. Cells deficient in iRhoms are resistant to ER stress-induced death.

Supplementary Figure 1. Cells deficient in iRhoms are resistant to ER stress-induced death. **a** Survival of WT and iRhom1/2 DKO MEFs was measured after 18 h and 36 h treatment with tunicamycin (0.5 µg/ml) by PI/Annexin V staining using flow cytometry. Percentages of live cells are expressed as mean ± SEM (n=3, biologically independent experiments). **b C**ell lysates of WT and iRhom1/2 DKO A549 cells after exposure to tunicamycin (2 µg/ml or 4 µg/ml) or brefeldin A (1 µg/ml or 2 µg/ml) for 18 h were analysed by immunoblotting with indicated antibodies. **c** Cell lysates of WT and iRhom2 KO BV2a cells after exposure to tunicamycin (0.25 µg/ml or 0.5 µg/ml, 18 h) were analysed by immunoblotting with indicated antibodies. **d** Cell lysates of WT, iRhom1/2 DKO MEFs after exposure to tunicamycin at indicated doses for 18 h were analysed by immunoblotting with indicated antibodies. Anti-KDEL antibody was used to detect both GRP94 and GRP78. **e** Change in level of *ero1L* transcripts was determined in WT and iRhom1/2 DKO MEFs treated with tunicamycin (0.5 µg/ml) for indicated time points by quantitative RT-PCR. Data are plotted relative to untreated cells for each genotype as mean ± SEM (n=3, biologically independent experiments). Immunoblotting data shown are representative of 2 biologically independent experiments. Source data are provided as a Source Data file.

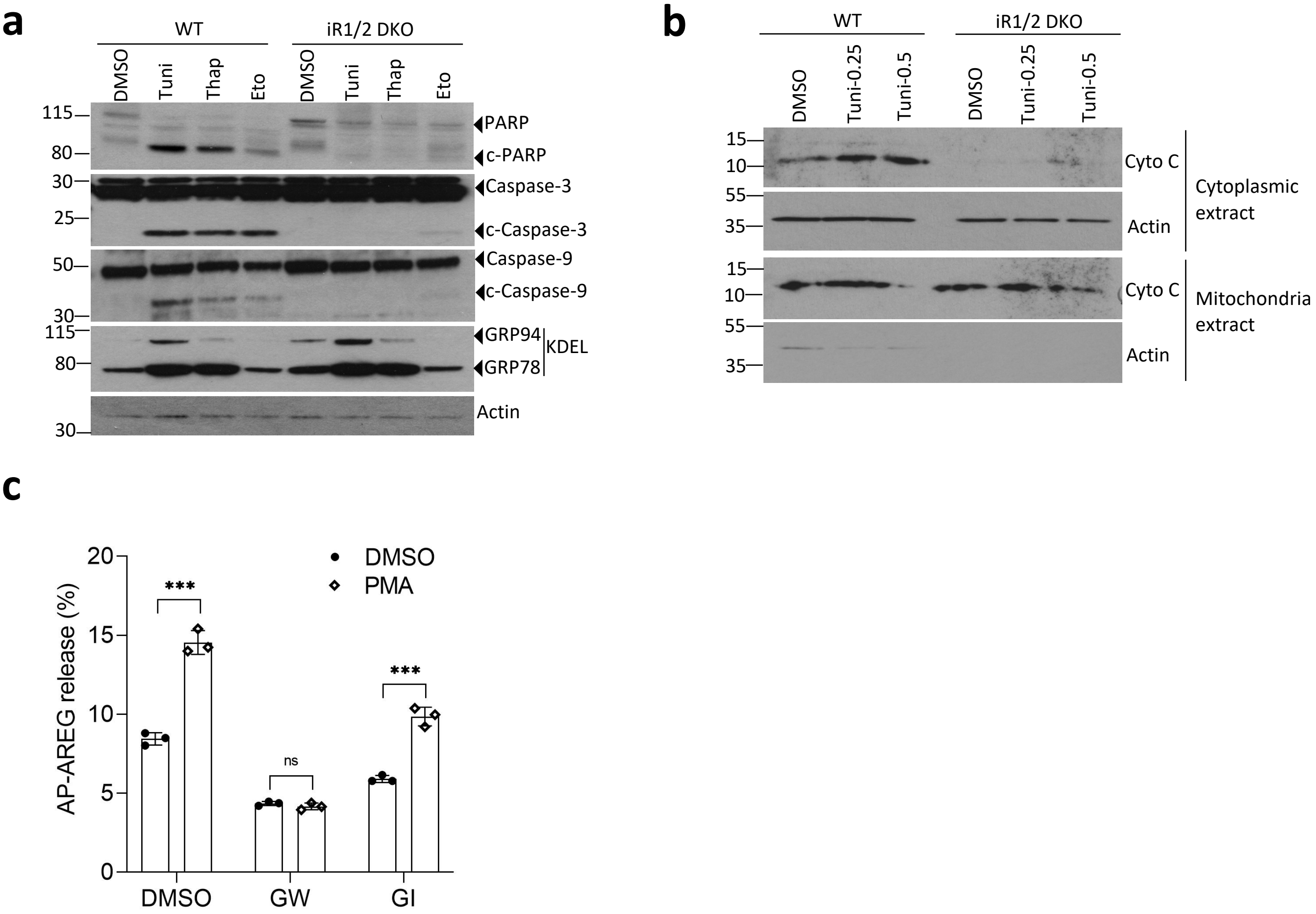

Supplementary Figure 2. iRhoms regulate the mitochondria-mediated cell death pathway

a Cell lysates of WT and iRhom1/2 DKO MEFs after exposure to tunicamycin (0.5 µg/ml), thapsigargin (1 µg/ml) or etoposide (25 µM) for 18 h were analysed by immunoblotting with indicated antibodies. **b** Levels of cytochrome c and actin were detected in fractionated cell lysates (cytoplasmic and mitochondria extracts) from WT and iRhom1/2 DKO MEFs exposed to tunicamycin (0.25 µg/ml and 0.5 µg/ml). **c** Bar chart shows shedding assay for alkaline-phosphatase tagged AREG after treatment with PMA (200 nM) for 30 min, in the presence of ADAM protease inhibitors GI254023X (5 µM; specific to ADAM10) and GW280264X (5 µM; inhibits both ADAM10 and ADAM17) (n=3 independent replicates). Two-way ANOVA (Sidak's), *** denotes p<0.001, ns denotes not significant. Immunoblotting data shown are representative of 2 biologically independent experiments. Source data are provided as a Source Data file.

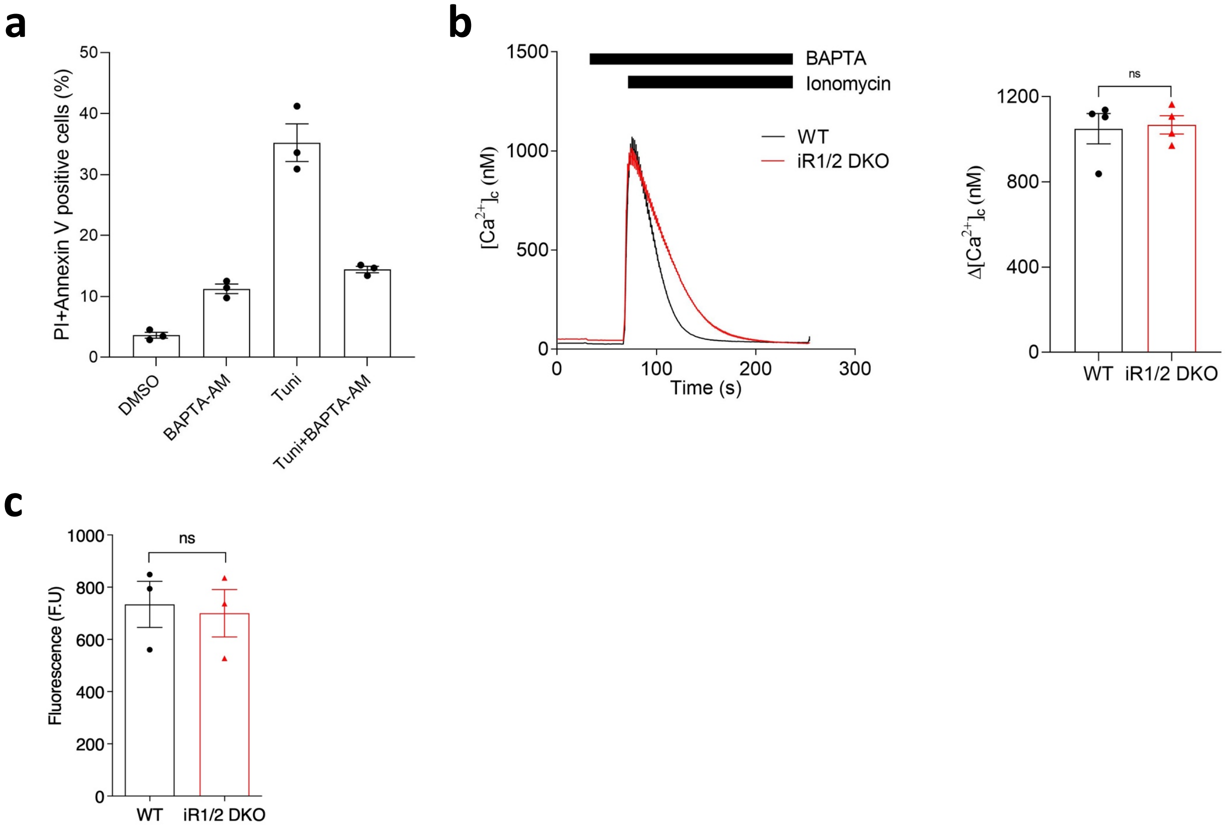

Supplementary Figure 3. ER stress-mediated cell death requires intracellular Ca^{2+} signalling. a Cell death of WT MEFs was measured after 18 h treatment with tunicamycin (0.5 µg/ml) and/or BAPTA-AM (50 µM) by PI/Annexin V staining using flow cytometry. Percentage of dead cells positive for both PI and Annexin V are shown in bar chart. Data are plotted as mean ± SEM (n=3, biologically independent experiments). **b** ER Ca^{2+} content was measured in populations of Calbryte 520-loaded WT and iRhom1/2 DKO MEFs stimulated with ionomycin (5µM) to release Ca^{2+} from intracellular stores. BAPTA (2.5 mM) was added 30 s prior to stimulation with ionomycin to chelate extracellular Ca^{2+}. Bar chart shows quantification of ER Ca^{2+} content, mean ± SEM, n=4 biologically independent experiments. Two-tailed paired Student's t test**,** ns= not significant. **c** WT and iR1/2 DKO MEFs were incubated with Calbryte-520 under exactly the conditions used for analyses of cytoplasmic [Ca^{2+}]. Cells were then lysed into HBS to determine the fluorescence of the Ca^{2+}-saturated Calbryte-520. FU, fluorescence units. Mean ± SEM, from n=3 biologically independent experiments, each with 12 replicates, Two-tailed paired Student's t test, ns: not significant. Source data are provided as a Source Data file.

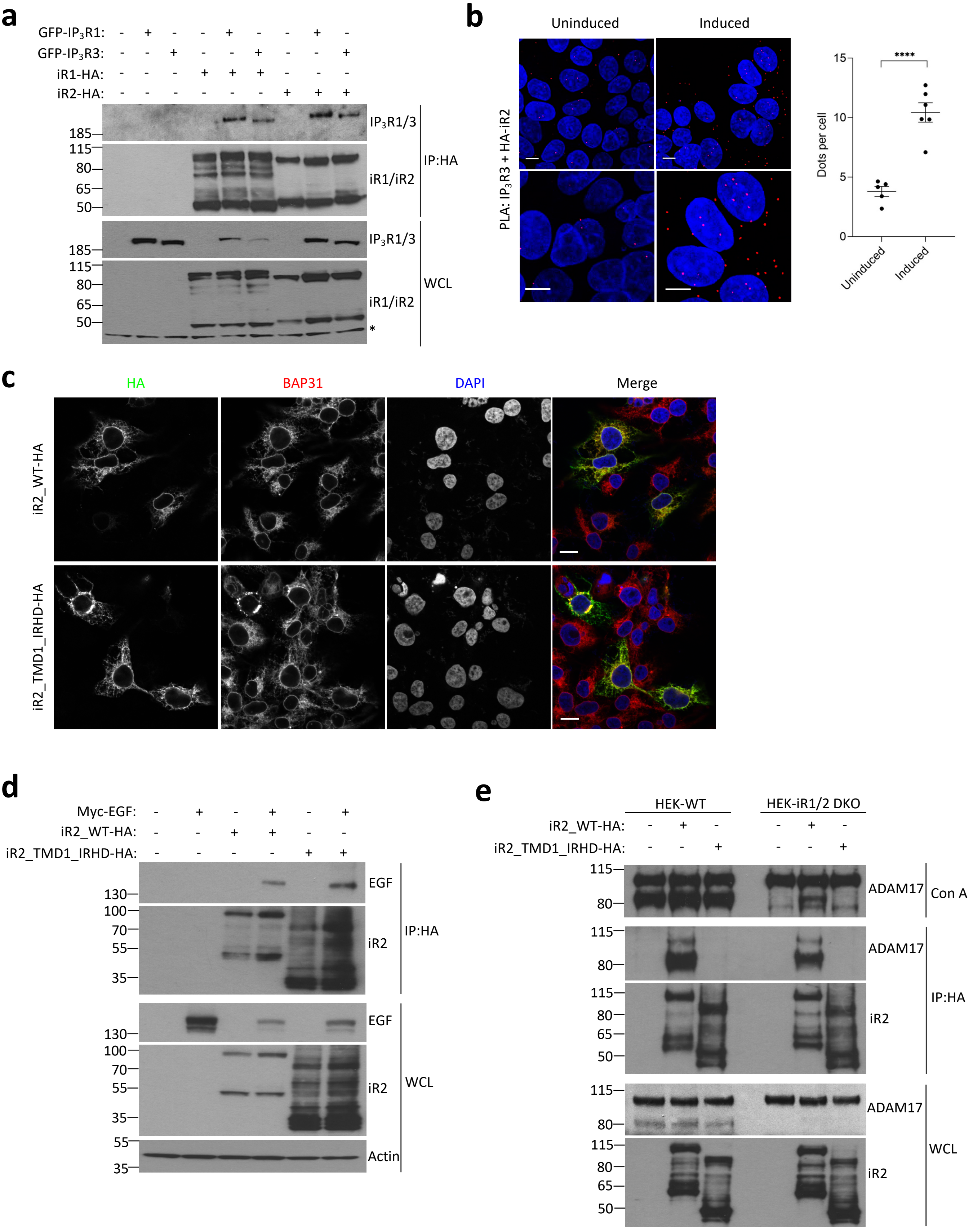

Supplementary Figure 4. IP$_3$Rs associate with iRhoms.

Supplementary Figure 4. IP_3Rs associate with iRhoms. a Levels of iRhom1, iRhom2, IP_3R1 and IP_3R3 were analysed by immunoblotting in whole cell lysate (WCL) and immunoprecipitation (IP: HA) from HEK293T cells transiently transfected for 36 h with GFP-IP_3R1 or GFP-IP_3R3 and iRhom1-HA or iRhom2-HA. * denotes unspecific band **b** Proximity ligation assay (PLA) in iRhom1/2 DKO HEK293T cells reconstituted with HA-iRhom2 under a tetracycline-inducible promoter were stained using antibodies to detect endogenous IP_3R3 and N-terminally HA-tagged iRhom2 after 24 h of 250 ng/ml of doxycycline to induce iRhom2 expression. Mean number of dots per cell were quantified using ImageJ and shown as mean ± SEM, uninduced (n=5 images/320 cells), induced (n=6 images/285 cells) from two biologically independent experiments. Two-tailed unpaired Student's t-test **** = $p<0.0001$. Scale bars = 10 μm. **c** Immunofluorescence of exogenously expressed iRhom2_WT-HA and iRhom2_TMD1_IRHD_HA transfected in HEK293T cells for 36 h. Cells were stained for HA (green), BAP31 as ER marker (red) and DAPI (blue). Scale bar =10 μm. Images are representative of 2 biologically independent experiments. **d** Levels of iRhom2_WT and iRhom2_TMD1_IRHD and EGF were determined by immunoblotting in whole cell lysate (WCL) and after immunoprecipitation (IP: HA) from HEK293T cells transiently transfected for 36hrs with Myc-EGF with iRhom2_WT-HA or iRhom2_WT_TMD1_IRHD-HA. **e** Maturation of ADAM17 using Concanavalin A (Con A) pull-down and immunoprecipitation (IP: HA) of ADAM17 in HEK293T-WT and HEK293T-iR1/2 DKO cells transfected for 36 h with iRhom2_WT -HA and iRhom2_TMD1_IRHD-HA. Immunoblotting data shown are representative of 2 biologically independent experiments. Source data are provided as a Source Data file.

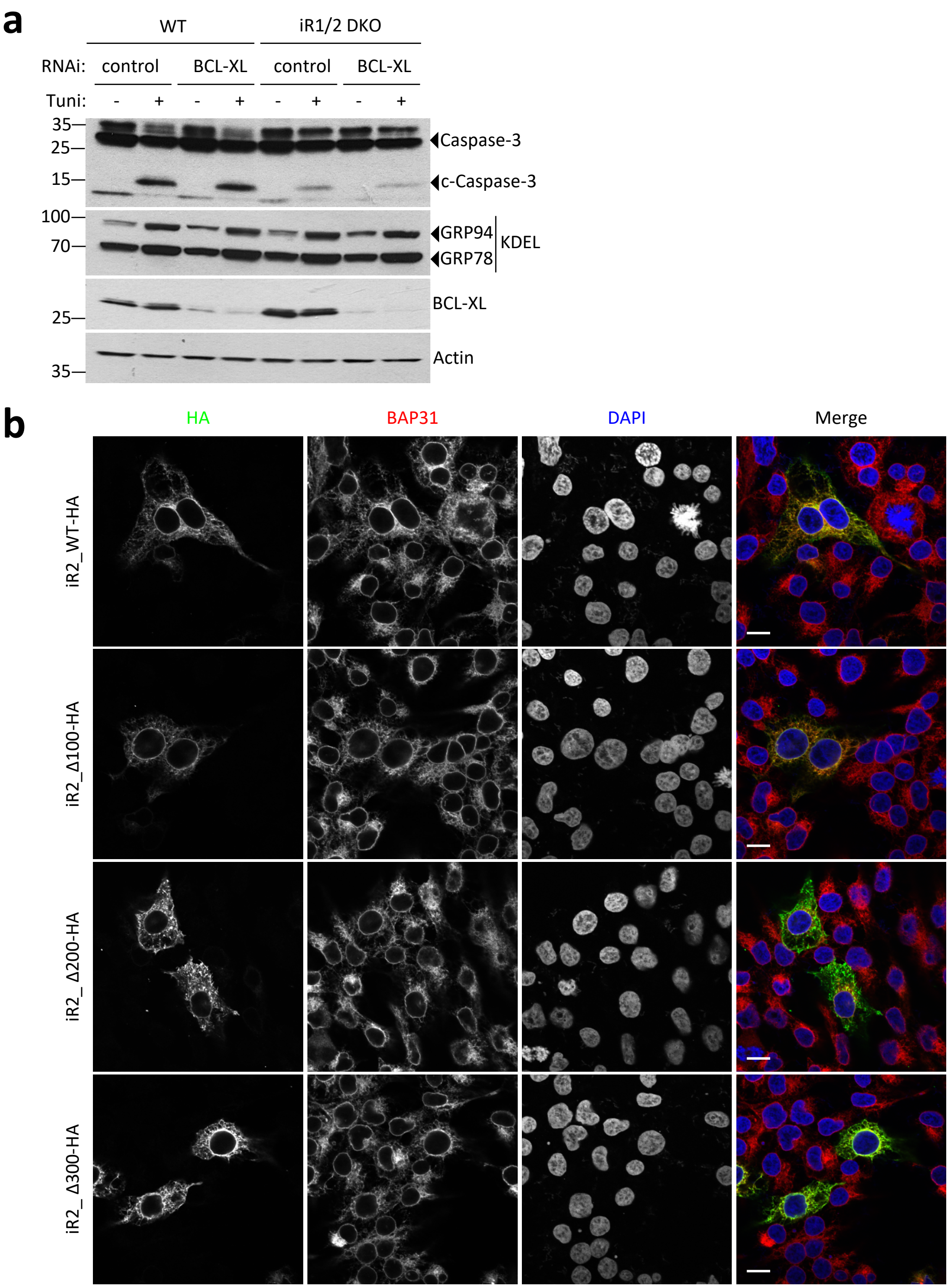

Supplementary Figure 5. Anti-apoptotic BCL-XL is not involved in iRhoms-regulated ER stress-induced cell death. a Cell lysates from WT and iRhom1/2 DKO MEFs transfected with control or *Bcl-xl* RNAi for 72 h, with tunicamycin (0.5 μg/ml) added in the last 18 h were analysed by immunoblotting with indicated antibodies. Immunoblotting data shown are representative of 2 biologically independent experiments. **b** Immunofluorescence of exogenously expressed iRhom2_WT-HA and indicated N-terminally truncated iRhom2 mutants transfected in HEK293T cells for 36 h. Cells were stained for HA (green), BAP31 as ER marker (red) and DAPI (blue). Scale bars = 10 μm. Images are representative of 2 biologically independent experiments. Source data are provided as a Source Data file.

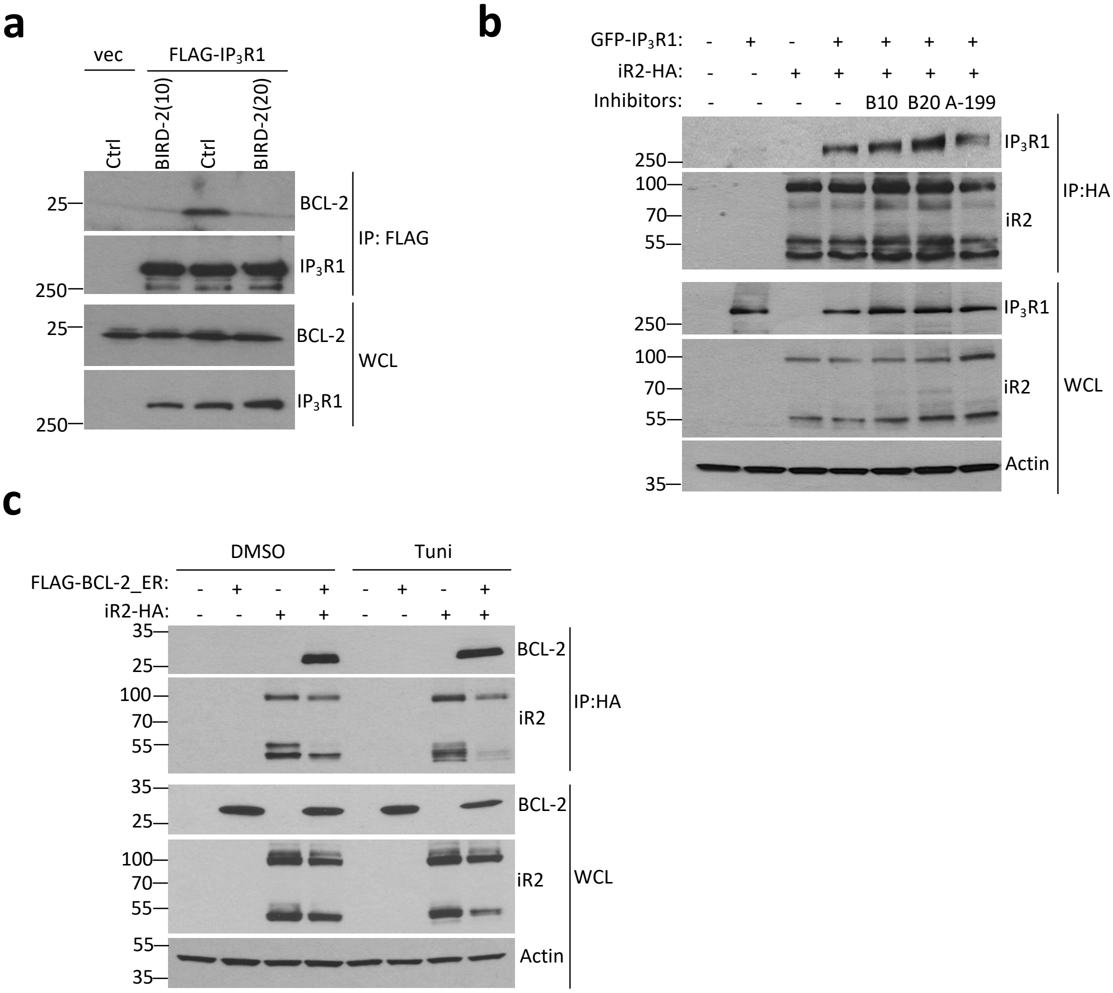

Supplementary Figure 6. BIRD-2 peptide increases association of iRhom2 with IP_3R1. a Levels of IP_3R1 and BCL-2 were analysed by immunoblotting in whole cell lysate (WCL) and after immunoprecipitation (IP: HA) from HEK293T cells transiently transfected with FLAG-IP_3R1 (2 µg) for 36 h in the presence of BIRD-2 (10 µM and 20 µM) for the last 18 h. **b** Levels of IP_3R1 and iRhom2 were analysed by immunoblotting in whole cell lysate (WCL) and after immunoprecipitation (IP: HA) from HEK293T cells transiently transfected with GFP-IP_3R1 (0.75 µg) and iRhom2-HA (0.25 µg) for 36 h in the presence of BIRD-2 (B10=10 µM or B20=20 µM) or ABT-199 (1 µM) added for the last 18 h. **c** Levels of iRhom2 and BCL-2 were analysed by immunoblotting in whole cell lysate (WCL) and after immunoprecipitation (IP: HA) from HEK293T cells transiently transfected with FLAG-BCL-2_ER and iRhom2-HA for 36hrs, with tunicamycin (2 µg/ml) added for last 18 h. Immunoblotting data shown are representative of 2 biologically independent experiments. Source data are provided as a Source Data file.

a

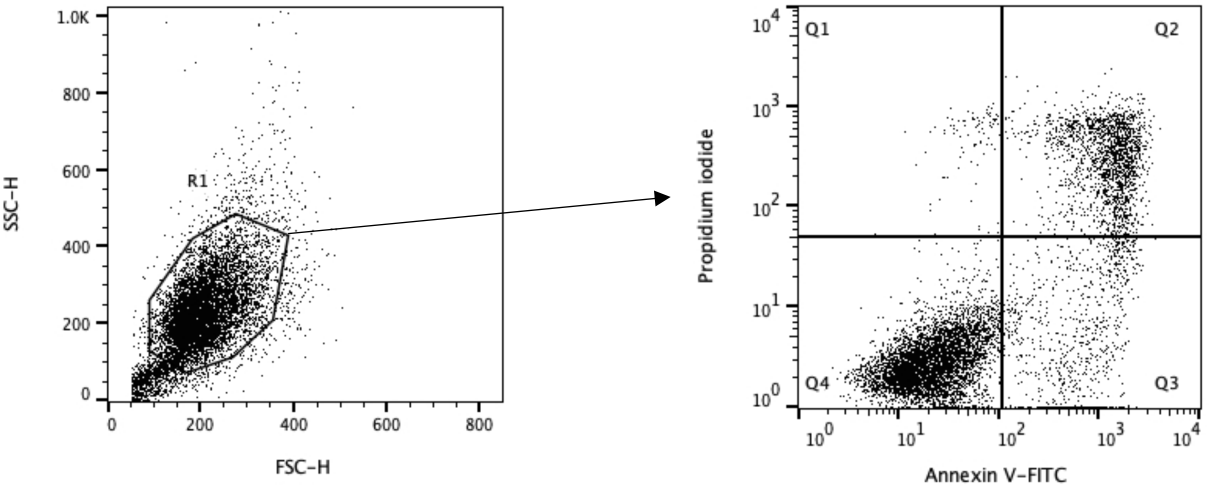

Supplementary Figure 7. Example of gating strategy used for PI/Annexin V-FITC staining to measure cell death. a Cell debris from original cell population were gated out from FSC-H and SSC-H plot denoted by region R1. Subset cell population R1 was then separated based on Annexin V-FITC and Propidium Iodide staining into 4 quadrants. Percentage of double-stained cells (quadrant Q2) was used as measurement of apoptosis.

Supplementary Table 1. Exact p-values for all statistically analysis

Fig. 1a	
WT v/s iR1/2 DKO	Exact p-value
DMSO	0.979
Tuni	<0.0001
Bref A	<0.0001
Fig. 1b	
WT v/s iR1/2 DKO	Exact p-value
DMSO	>0.9999
Tuni-0.25	<0.0001
Tuni-0.5	0.0004
Bref A	0.001
Fig. 2d	
WT v/s iR1/2 DKO	Exact p-value
Tuni-0.25	<0.0001
Tuni-0.5	<0.0001
Bref A	0.0012
FCCP	0.9469
Fig. 2e	
WT v/s iR1/2 DKO	Exact p-value
DMSO/GI/GW	<0.0001
Fig. 3a	
WT v/s iR1/2 DKO	Exact p-value
ATP	0.0001
Fig. 3b	
WT v/s iR1/2 DKO	Exact p-value
BK	0.0001
Fig. 3c	
WT v/s iR1/2 DKO	Exact p-value
BAPTA/TG	0.1225
Fig. 3d	
WT v/s iR1/2 DKO	Exact p-value
IP3	0.0114
Fig. 3e	
WT v/s iR1/2 DKO	Exact p-value
caged-IP3	0.019
Fig. 3f	
Cells	Exact p-value
WT v/s iR1/2 DKO	0.0016
WT v/s WT+2-APB	0.0008
WT v/s WT+Xest-C	0.0228
Fig. 3g	
WT v/s iR1/2 DKO	Exact p-value
Ctrl+DMSO	0.1034
Tuni+DMSO	<0.0001
Ctrl+2-APB	0.828
Tuni+2-APB	0.4692
Fig. 4c	
Cells	Exact p-value
Unind. vs Induced	<0.0001
Fig. 4f	
Cells	Exact p-value
DMSO vs Tuni	0.0078

Fig. 5a	
WT v/s iR1/2 DKO	Exact p-value
Itpr1	0.0013
Itpr2	0.9816
Itpr3	0.8881
Fig. 5c (protein)	
WT v/s iR1/2 DKO	Exact p-value
BCL-2	<0.0001
BCL-XL	0.0002
Fig. 5c (RNA)	
WT v/s iR1/2 DKO	Exact p-value
Bcl-2	0.0038
Bcl-xl	0.0863
Fig. 5d	
Ctrl RNAi - BCL-2 RNAi	Exact p-value
WT	0.3067
iR1/2 DKO	0.0077
Suppl. Fig. 2c	
DMSO v/s PMA	Exact p-value
DMSO	<0.0001
GW	0.9434
GI	<0.0001
Suppl. Fig. 3b	
Cells	Exact p-value
WT v/s iR1/2 DKO	0.8584
Suppl. Fig. 3c	
Cells	Exact p-value
WT v/s iR1/2 DKO	0.8013
Suppl. Fig. 4b	
Cells	Exact p-value
Unind v/s induced	<0.0001

Supplementary Table 2. Primer sequences used for cloning

Primers	Sequence	Purpose
miR1-BglII-Fwd	5' AAGCTTGCCAAGATCTATGAGTGAAGCCAGGAGAGAC 3'	Cloning of 3XFLAG C-term tagged mouse iR1 into pM6P vector
miR1-NotI-Rev	5' GGAGAGGGGCGGCCGCTCACTTGTCATCGTCATCCT 3'	Cloning of 3XFLAG C-term tagged mouse iR1 into pM6P vector
miR2(-100)-BglII-Fwd	5' TACCGGACTCAGATCTATGGACTGGGAGGGCAAGCGACAA 3'	Cloning of 3XHA C-term tagged mouse iR2 deletion mutant into pEGFP-N1 vector
miR2(-200)-BglII-Fwd	5' TACCGGACTCAGATCTATGCATCTGCCCCGCCGCAAGAGG 3'	Cloning of 3XHA C-term tagged mouse iR2 deletion mutant into pEGFP-N1 vector
miR2(-300)-BglII-Fwd	5' TACCGGACTCAGATCTATGGGAGTGCACATCCCGCTAAAA 3'	Cloning of 3XHA C-term tagged mouse iR2 deletion mutant into pEGFP-N1 vector
miR2-NotI-Rev	5' TCTAGAGTCGCGGCCGCTTAGCCGGCGTAATCCGGCA 3'	Cloning of 3XHA C-term tagged mouse iR2 deletion mutant into pEGFP-N1 vector
miR2-BglII-Fwd	5' TACCGGACTCAGATCTATGGCCTCAGCTGACAAGAAT 3'	Cloning of 3XHA C-term tagged mouse iR2 deletion mutant into pEGFP-N1 vector
miR2_TMD1+IRHD_XhoI-Rev	5' GGTATGCACCCTCGAGCCGGTAGAACTGGTCAGGGAC 3'	Cloning of 3XHA C-term tagged mouse iR2 deletion mutant into pEGFP-N1 vector
