# Supplementary material for: iRhom pseudoproteases regulate ER stress-induced cell death through IP3 receptors and BCL-2
Source: Nat Commun. 2022 Mar 10;13:1257. doi: 10.1038/s41467-022-28930-4 (PMC8913617; doi:10.1038/s41467-022-28930-4)
Supplement: Supplementary file 3 — Reporting Summary [file 41467_2022_28930_MOESM3_ESM.pdf]

nature research

Corresponding author(s): Iqbal Dulloo and Matthew Freeman,
Last updated by author(s): 14/01/22

Reporting Summary

Nature Research wishes to improve the reproducibility of the work that we publish. This form provides structure for consistency and transparency in reporting. For further information on Nature Research policies, see our Editorial Policies and the Editorial Policy Checklist.

Statistics

For all statistical analyses, confirm that the following items are present in the figure legend, table legend, main text, or Methods section.

n/a	Confirmed	
☐	☒	The exact sample size (*n*) for each experimental group/condition, given as a discrete number and unit of measurement
☐	☒	A statement on whether measurements were taken from distinct samples or whether the same sample was measured repeatedly
☐	☒	The statistical test(s) used AND whether they are one- or two-sided<br>*Only common tests should be described solely by name; describe more complex techniques in the Methods section.*
☒	☐	A description of all covariates tested
☐	☒	A description of any assumptions or corrections, such as tests of normality and adjustment for multiple comparisons
☐	☒	A full description of the statistical parameters including central tendency (e.g. means) or other basic estimates (e.g. regression coefficient) AND variation (e.g. standard deviation) or associated estimates of uncertainty (e.g. confidence intervals)
☐	☒	For null hypothesis testing, the test statistic (e.g. *F*, *t*, *r*) with confidence intervals, effect sizes, degrees of freedom and *P* value noted<br>*Give P values as exact values whenever suitable.*
☒	☐	For Bayesian analysis, information on the choice of priors and Markov chain Monte Carlo settings
☒	☐	For hierarchical and complex designs, identification of the appropriate level for tests and full reporting of outcomes
☒	☐	Estimates of effect sizes (e.g. Cohen's *d*, Pearson's *r*), indicating how they were calculated

Our web collection on statistics for biologists contains articles on many of the points above.

Software and code

Policy information about availability of computer code

Data collection	Metamorph v7.10.1.161, SoftMax Pro v5.4, StepOnePlus v2.0, BD CellQuest Pro v5.1
Data analysis	Metamorph v7.10.1.161, SoftMax Pro v5.4, GraphPad Prism v6, Fiji v1.5n, IGOR Pro v7.0.8.1, FlowJo v10.8

For manuscripts utilizing custom algorithms or software that are central to the research but not yet described in published literature, software must be made available to editors and reviewers. We strongly encourage code deposition in a community repository (e.g. GitHub). See the Nature Research guidelines for submitting code & software for further information.

Data

Policy information about availability of data

All manuscripts must include a data availability statement. This statement should provide the following information, where applicable:

- Accession codes, unique identifiers, or web links for publicly available datasets
- A list of figures that have associated raw data
- A description of any restrictions on data availability

Authors can confirm that all relevant data supporting the conclusions of this paper are included in the main figures and the supplementary information files. Source data are provided as Source Data file. Data availability statement has been included in the manuscript.

Field-specific reporting

Please select the one below that is the best fit for your research. If you are not sure, read the appropriate sections before making your selection.

[x] Life sciences [ ] Behavioural & social sciences [ ] Ecological, evolutionary & environmental sciences

For a reference copy of the document with all sections, see nature.com/documents/nr-reporting-summary-flat.pdf

Life sciences study design

All studies must disclose on these points even when the disclosure is negative.

Sample size	No predetermined samples size were used. Smallest number of independent replicates reported are n=2 for some western blots, with most n=3 or more, both which have historically provided sufficient consistency to support the conclusions presented. It was impracticable to complete formal power analyses before deciding sample sizes. All sample sizes were dictated by feasibility and where the outcomes of experiments were quantified, sufficient replicates were performed to allow statistical analyses of the data (at least n=3).
Data exclusions	No data were excluded
Replication	Replications for each data in paper are indicated in figure legends In brief:<br>All western blots data were replicated 2-3 times each.<br>Data for cell death assays and mitochondrial membrane potential were replicated at least 3 times.<br>All calcium measurements were replicated at least 3 times.<br>All qPCR data were replicated 3 times.<br>All immunofluorescence data were replicated at least 2 times.
Randomization	There was no need for randomization of samples as most treatments were done in parallel for each cell lines with the treatments as the only variable. Observer bias in analysis of images was avoided by quantitative analysis
Blinding	Blinding was not required for this study because there were no data exclusions and conclusions were made on objective quantitative analysis of data

Reporting for specific materials, systems and methods

We require information from authors about some types of materials, experimental systems and methods used in many studies. Here, indicate whether each material, system or method listed is relevant to your study. If you are not sure if a list item applies to your research, read the appropriate section before selecting a response.

Materials & experimental systems

n/a	Involved in the study	
[ ]	[x]	Antibodies
[ ]	[x]	Eukaryotic cell lines
[x]	[ ]	Palaeontology and archaeology
[ ]	[x]	Animals and other organisms
[x]	[ ]	Human research participants
[x]	[ ]	Clinical data
[x]	[ ]	Dual use research of concern

Methods

n/a	Involved in the study	
[x]	[ ]	ChIP-seq
[ ]	[x]	Flow cytometry
[x]	[ ]	MRI-based neuroimaging

Antibodies

Antibodies used	For immunoblotting and co-immunoprecipitation: Actin (Santa Cruz, sc-47778; 1:5000), ATF6 (Cell Signaling Technology, 65880; 1:1000), BCL-2 (AbCam, ab182858; 1:500), BCL-XL (Cell Signaling Technology, 2764; 1:1000), Cleaved-Caspase-8 (Cell Signaling Technology, 8592; 1:1000), Caspase-3 (Cell Signaling Technology, 9665; 1:1000), Caspase-9 (Cell Signaling Technology, 9508; 1:1000), CHOP (Cell Signaling Technology, 2895; 1:250), GFP (AbCam, ab13970; 1:2000), GRP78 (Cell Signaling Technology, 3177; 1:1000), FLAG-HRP (Sigma Aldrich, A8592; 1:4000), HA-HRP (Roche, 11867423001; 1:2000), IP3R1 (Thermo Fischer, PA1-901; 1:1000), IP3R1 & IP3R2 [previously described 77; 1:1000], IP3R3 (BD Biosciences, 610312; 1:1000), KDEL (AbCam, ab12223; 1:2000), Mcl-1 (Cell Signaling Technology, 5453; 1:1000), Nicastrin (BD Biosciences, 612290; 1:1000), PARP (Cell Signaling Technology, 9542; 1:1000), SERCA2 (Cell Signaling Technology, 4388; 1:1000), STIM1 (Cell Signaling Technology, 5668; 1:1000).<br>For immunofluorescence: DAPI (Thermo Fischer, D1306; 1 µg/ml), HA (Cell Signaling Technology, 3724; 1:500), BAP31 (Enzo Life Sciences, ALX-804-601-C100; 1:250).<br>For proximity ligation assay: HA (Enzo Life Sciences, ALX-804-601-C100; 1:200), HA (Enzo Life Sciences, ENZ-ABS118-0200; 1:200), IP3R1 (Thermo Fischer, PA1-901; 1:200), IP3R3 (BD Biosciences, 610312; 1:200).

Validation	All commercial antibodies were chosen based on the validation on manufacturer's website and if they are supported by multiple citations; and reference papers are provided for non-commercial antibodies.<br><br>Western Blots<br>Actin: https://www.scbt.com/p/beta-actin-antibody-c4<br>ATF6: https://www.cellsignal.com/products/primary-antibodies/atf-6-d4z8v-rabbit-mab/65880<br>BCL-2: https://www.abcam.com/Bcl-2-antibody-EPR17509-ab182858.html<br>BCL-XL: https://www.cellsignal.co.uk/products/primary-antibodies/bcl-xl-54h6-rabbit-mab/2764<br>Cleavead caspase-8: https://www.cellsignal.co.uk/products/primary-antibodies/cleaved-caspase-8-asp387-d5b2-xp-rabbit-mab-mouse-specific/8592<br>caspase-3: https://www.cellsignal.co.uk/products/primary-antibodies/caspase-3-8g10-rabbit-mab/9665<br>caspase-9: https://www.cellsignal.co.uk/products/primary-antibodies/caspase-9-c9-mouse-mab/9508<br>CHOP: https://www.cellsignal.co.uk/products/primary-antibodies/chop-l63f7-mouse-mab/2895<br>GFP: https://www.abcam.com/GFP-antibody-ab13970.html<br>GRP78: https://www.cellsignal.co.uk/products/primary-antibodies/bip-c50b12-rabbit-mab/3177<br>FLAG-HRP: https://www.sigmaaldrich.com/GB/en/product/sigma/a8592<br>HA-HRP: https://www.sigmaaldrich.com/GB/en/product/roche/roahaha<br>IP3R1: https://www.thermofisher.com/antibody/product/IP3-Receptor-1-Antibody-Polyclonal/PA1-901<br>IP3R2: Reference 77 in manuscript<br>IP3R3: https://www.bdbiosciences.com/en-eu/products/reagents/microscopy-imaging-reagents/immunofluorescence-reagents/purified-mouse-anti-ip3r-3.610312<br>KDEL: https://www.abcam.com/kdel-antibody-10c3-ab12223.html<br>MCL-1: https://www.cellsignal.co.uk/products/primary-antibodies/mcl-1-d35a5-rabbit-mab/5453<br>Nicastrin: https://www.bdbiosciences.com/en-eu/products/reagents/western-blotting-and-molecular-reagents/western-blot-reagents/purified-mouse-anti-nicastrin.612290<br>PARP: https://www.cellsignal.co.uk/products/primary-antibodies/parp-antibody/9542<br>SERCA2: https://www.cellsignal.co.uk/products/primary-antibodies/atp2a2-serca2-antibody/4388<br>STIM1: https://www.cellsignal.co.uk/products/primary-antibodies/stim1-d88e10-rabbit-mab/5668<br><br>Immunofluorescence<br>HA: https://www.cellsignal.co.uk/products/primary-antibodies/ha-tag-c29f4-rabbit-mab/3724<br>BAP31: https://www.enzolifesciences.com/ALX-804-601/bap31-monoclonal-antibody-a1-182/<br><br>Proximity Ligation assay<br>HA:https://www.cellsignal.co.uk/products/primary-antibodies/ha-tag-c29f4-rabbit-mab/3724<br>HA: https://www.enzolifesciences.com/ENZ-ABS118/ha.11-monoclonal-antibody-16b12/<br>IP3R1: https://www.thermofisher.com/antibody/product/IP3-Receptor-1-Antibody-Polyclonal/PA1-901<br>IP3R3: https://www.bdbiosciences.com/en-eu/products/reagents/microscopy-imaging-reagents/immunofluorescence-reagents/purified-mouse-anti-ip3r-3.610312

Eukaryotic cell lines

Policy information about cell lines

Cell line source(s)	All mouse embryonic fibroblasts (MEFs) lines used were previously generated in the lab (Science 335, 225-228, 2012)<br>A549 cells: adenocarcinomic human alveolar basal epithelial cells (Fodor Lab, Oxford)<br>HEK293T cells: human embryonic kidney 293 cells containing the SV40 T-antigen (originally from ATCC)<br>BV2a cells: mouse microglia cells (originally from ATCC)
Authentication	HEK293T and BV2a cells were bought commercially via ATCC, which already include authentication. A549 and MEFs (generated in house) were not authenticated.
Mycoplasma contamination	All cell lines were routinely screened and were mycoplasma-free
Commonly misidentified lines<br>(See ICLAC register)	No commonly misidentified cell lines used in this study

Animals and other organisms

Policy information about studies involving animals; ARRIVE guidelines recommended for reporting animal research

Laboratory animals	4-week old female Drosophila melanogaster were used. No mice were used or bred for this study. Mouse embryonic fibroblasts used were previously generated in lab (Science 335, 225-228, 2012).
Wild animals	This study did not involve wild animals

Field-collected samples	This study did not involve samples collected from field
Ethics oversight	No ethical approval or guidance was required in this study, which is mainly done in cell lines. Drosophila melanogaster studies do not required any ethic oversight as per regulation of University of Oxford.

Note that full information on the approval of the study protocol must also be provided in the manuscript.

Flow Cytometry

Plots

Confirm that:

- [x] The axis labels state the marker and fluorochrome used (e.g. CD4-FITC).
- [x] The axis scales are clearly visible. Include numbers along axes only for bottom left plot of group (a 'group' is an analysis of identical markers).
- [x] All plots are contour plots with outliers or pseudocolor plots.
- [x] A numerical value for number of cells or percentage (with statistics) is provided.

Methodology

Sample preparation	MEFs cells were used for all experiments involving FACS analysis. Cells were harvested after incubation with indicated dyes, washed in PBS and processed according to respective manufacturer's protocols indicated in Methods section
Instrument	BD FASCCalibur
Software	BD CellQuest Pro v5.1 was used for data acquisition and FlowJo v10.8 was used for data analysis
Cell population abundance	Acquisition was done at same flow rate for number of events. For cell death assays, 10,000 cells were acquired for each sample. For TMRE staining, 30,000 cells were acquired for each sample. Cell debris were gated out, leaving around 80-90% cells, which were used for analysis.
Gating strategy	Cell debris/clumps from original population were first gated out using FSC/SSC. Unstained cells were used as reference to mark areas where cells will be positive for dyes used. Strategy is illustrate in Suppl. Fig. 7

- [x] Tick this box to confirm that a figure exemplifying the gating strategy is provided in the Supplementary Information.
